# The Sea Was Angry That Day My Friends: An Inland Case of Acute Scombroid Poisoning With a Twist

**DOI:** 10.7759/cureus.18394

**Published:** 2021-09-30

**Authors:** Roger Taylor, Joshua Burg, Nnennaya Opara

**Affiliations:** 1 Emergency Medicine, Charleston Area Medical Center, Charleston, USA; 2 Emergency Medicine, Charleston Area Medical Center Health Education and Research Institute, Charleston Area Medical Center, Charleston, USA

**Keywords:** diarrhea, rash, tachycardia, antihistamine, fish, ekg, poisoning, anaphylaxis, histamine, scombroid

## Abstract

Scombroid poisoning is a common form of food poisoning related to fresh, canned, or smoked fish ingestion with high histamine content as a result of improper handling and storage. The incubation period for this type of fish poisoning is relatively short (ranges from a few minutes to hours). We present a case of a 53-year-old male who developed severe symptoms of scombroid poisoning minutes after ingesting an ahi tuna salad in a local restaurant.

## Introduction

Scombroid poisoning is a condition that is very uncommon and rarely seen in coastal areas. It is most frequently implicated in the consumption of dark meat fish but can also be associated with other groups of fish all of which contain high levels of free histidine from improper handling resulting in bacterial overgrowth that causes the conversion of histidine to histamine [1]. It was initially thought that this process only resulted from the consumption of fish from the scombroid family. However, various families of fish and cheeses that contain elevated levels of histamine and other products of decomposition have been implicated [2]. The fish itself must be stored at temperatures less than 40 degrees Fahrenheit from catch until it is prepared for human consumption to avoid an irreversible chemical process that results in histamine formation which may occur even with cooking, so prevention with proper handling is key [3]. A typical presentation of scombroid poisoning usually involves a relatively rapid onset of symptoms within 20-30 minutes and lasts for 4-48 hours [3]. The cause appears to be somewhat simple on the surface but may also be a complex interplay of factors.

Common symptoms such as erythematous rash, flushing of the skin, headache, and diarrhea frequently occur. Patients can additionally have abdominal cramps, blurred vision, nausea, and sweating. There is a concern in these presentations for overlap with anaphylaxis and the clinician should be aware to look for evidence of airway edema, bronchospasm, tongue swelling, respiratory distress, or hypotension [3]. Patients typically improve rather quickly with antihistamine medications and the overall prognosis is very good without chronic sequelae in the vast majority of cases [3].

Histamine has classically been implicated in scombroid poisoning. Histidine decarboxylase, found in bacteria such as Proteus, Escherichia coli, Clostridium, and Pseudomonas that live in fish gills and GI tracts, converts histidine to histamine [4]. Histamine, once formed, is extremely thermostable, and as discussed earlier, can cause symptoms despite cooking. Interestingly, in some cases of “histamine poisoning” measured serum histamine levels have been low. Other mechanisms have been considered including proteins such as cadaverine and putrescine acting as histamine activators, mast cell degranulation mechanisms, histamine receptor agonists, and individual histamine intolerance [5].

## Case presentation

In this case, a 53-year-old male presented by emergency medical services (EMS) with the chief complaint of nausea, vomiting, diarrhea that had all began abruptly around one hour prior to arrival. He additionally noted a developing headache and thought that his skin was more flushed than normal. The patient stated that he was in his normal state of health that morning and then had gone for lunch at a restaurant where he ate an ahi tuna salad. He stated that the food had a somewhat “peppery” taste to him when he took the first bite but finished the meal. He then began to feel ill around 10-15 minutes after eating with significant nausea and abdominal cramping. He had voluminous non-bloody diarrhea and developed a headache with diaphoresis and fatigue. His initial vital signs showed he was afebrile, tachycardic at 130 beats per minute, hypotensive at 88/57 mmHg, and tachypneic at 24 breaths per minute. He denied any chest pain, shortness of breath, and significant cardiac or medical history. He denied any recent travel or contact with other people with similar symptoms.

**Figure 1:**
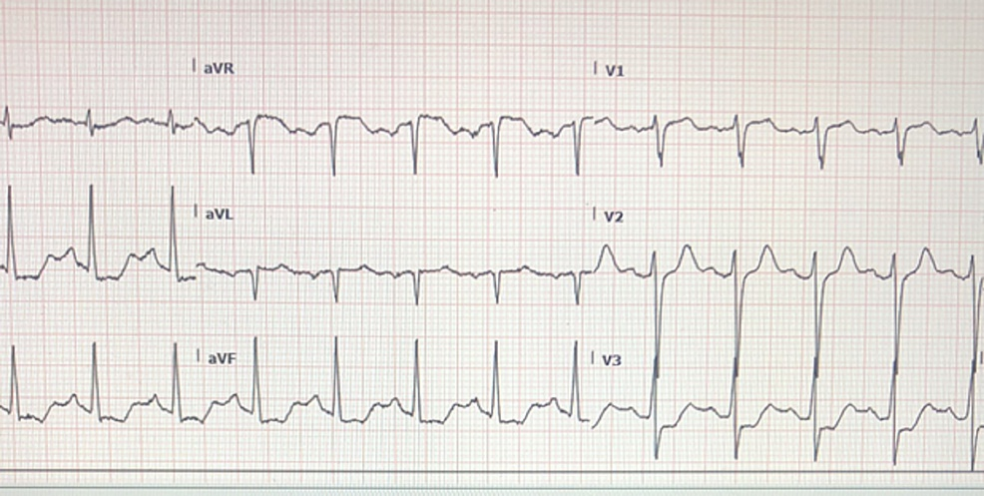
Initial EKG performed showing sinus tachycardia at 130 bpm with ST-segment depression in the infero-lateral lead.

On physical exam, the patient presented with tachycardia, diaphoresis, and flushing of his skin. The patient continued to complain of mild nausea with a warm sensation. The patient was administered a one-liter bolus of normal saline and an EKG was done which showed sinus tachycardia with ST depression in the inferio-lateral leads and borderline elevation in aVR (Figure 1). The patient remained without any complaints of chest pain. Lab workup showed slightly decreased hematocrit and potassium levels with elevated serum lactate, glucose, blood urea nitrogen (BUN), and creatinine levels (Table 1). Acute kidney injury was suspected and myocardial infarction was ruled out following a normal troponin test.

**Table 1:** Laboratory findings on admission. BUN: Blood urea nitrogen.

Blood serum, electrolyte	Patient	Normal range
WBC	6.9 cells/mcL	4.5-11 cells/mcl
Hemoglobin	13.8 g/dl	13.5-17.5 g/dl
Hematocrit	40.3%	41%-53%
Platelets	271,000/ mm^3^	150,000-450,000/mm^3^
Sodium	140 mmol/L	136-146 mmol/L
Potassium	3.5 mmol/L	3.5-5.0 mmol/L
Chloride	103 mmol/L	95-105 2mmol/L
Lactate	3.0 mmol/L	0.5-2.0 mmol/L
Glucose	211 mg/dl random	<140 mg/dl random
BUN	20 mg/dl	7-18 mg/dl
Creatinine	1.4 mg/dl	0.6-1.2mg/dl

**Figure 2:**
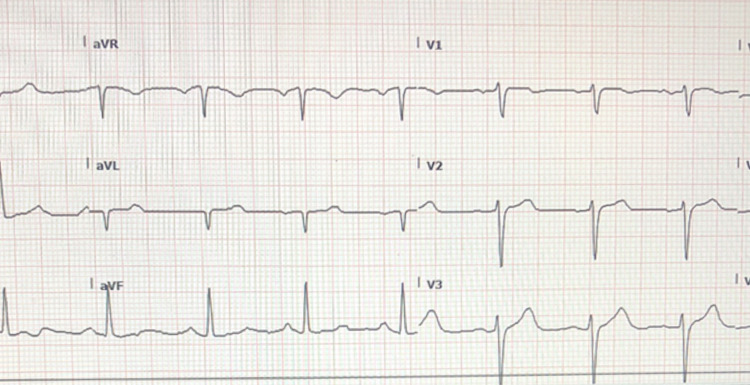
Repeat EKG performed approximately 45 minutes after treatment showing sinus rhythm at 80 bpm with resolution of ST depression as seen prior.

Differential diagnoses at this time included the possibility of anaphylaxis, allergic reaction/seafood allergy, acute coronary syndrome, staphylococcal food poisoning, ciguatera toxicity, or scombroid poisoning. Epinephrine was ordered even though the patient had no respiratory complaints or compromise and no airway edema or swelling. Steroids were administered with H1 and H2 blockers and 2 L normal saline for hydration. After 15-20 minutes, the patient’s symptoms began to abate. His flushing resolved and tachycardia decreased to around 100 bpm. He continued to improve significantly over the next half hour with a resolution of tachycardia and pruritus. Repeat EKG was performed with essential normalization of ST segments while the second troponin measurement was also normal and the patient remained chest pain-free (Figure 2). The patient did have a mild headache that continued but otherwise felt back to his baseline. Due to the alarming initial presentation with abnormal EKG, the patient was admitted and monitored overnight. His acute kidney injury resolved, he had no chest pain episodes, glucose normalized, and he was discharged after 24 hours. The restaurant where the patient visited was also informed about the poisoning.

## Discussion

Differential considerations include another frequent marine ingestion of ciguatera which is a marine toxin found in large reef fish that is predominantly concentrated in the Caribbean but has also been seen in areas near the Gulf of Mexico. It occurs from a species of plankton found on coral reefs and the toxins are not destroyed by cooking or freezing. Fifty percent of cases will have “temperature reversal” where cold objects feel warm or vice versa which is pathognomonic and differentiates it from scombroid poisoning [6]. Patients more frequently, but still rarely, will have hypotension and bradycardia with ciguatera. Some similarities exist with GI symptoms and pruritus. Another differentiating factor is often the delayed onset of symptoms from a few minutes to 12 hours as opposed to a rapid onset of scombroid [6].

In this case, there were some cardiac changes on the initial EKG which were concerning during the initial presentation (Figure 1]. The patient had diaphoresis and hypotension but was an active, non-smoking individual without known coronary heart disease or other obvious risk factors, and had no complaints of chest pain. Consideration in this setting was given to the possibility of an allergic syndrome causing cardiac manifestations. Most case reports of Kounis syndrome, or allergic angina, often involved patients with more underlying cardiovascular risk factors as opposed to the patient in our case [7]. This syndrome itself is proposed to occur via mast cell degranulation with the release of histamine and various inflammatory mediators. This is thought to result in coronary vasospasm. Another considered pathway includes tryptase release that can result in a cascade causing plaque rupture leading to thrombus. There are three types of Kounis syndrome. Type I is chest pain due to coronary artery vasospasm in patients without known cardiac disease. Type II occurs in patients that have an underlying coronary disease with vasospasm or plaque rupture resulting in myocardial infarction. In Type III disease, patients have coronary thrombi which have eosinophils and mast cells present [7]. Our patient did not definitively meet these criteria but would be most likely seem to be a type I picture. The patient was asymptomatic and did not receive a cardiac catheterization so it is impossible to completely know the patient’s underlying cardiovascular risk or presence of underlying coronary artery disease. The patient’s EKG was significant for ST depression and was concerning on initial presentation, but had significant improvement and essential resolution of EKG abnormalities within one hour after treatment of histamine surge (Figure 2). His troponins remained negative. Typical management involves histamine blockers and corticosteroids (such as in our case), and epinephrine to help improve vasospasm, and then classic acute coronary syndrome management as typically indicated. One caveat is that beta-blockers may decrease the effect of epinephrine and should be used cautiously in such settings [7].

## Conclusions

Scombroid poisoning is an underdiagnosed toxicity associated with both scombroid and non-scombroid fish. We present a case involving such a presentation in an inland location where less awareness of this disease process exists. This case demonstrates the importance of keeping a broad differential on initial presentation including anaphylaxis and cardiac etiologies. Once suspected, the local health department and locations associated with the poisoning should be contacted to discard potentially contaminated food. We should all be more aware of this disease process regardless of location for the sake of public health safety.
